# Progress in Medicine

**Published:** 1900-06-02

**Authors:** 


### PROGRESS IN MEDICINE.

### DISEASES OF THE BLOOD.

(*Continued from page* 137.)

**Leukæmia.**—A case of lymphatic leukæmia is reported by M. H. Spencer[11] noticeable for its acute course, the patient dying in two months from the time when he was first seen. There were also present large numbers of nucleated red cells, which are rarely seen in this form of leukæmia. Both the spleen and liver were greatly enlarged, and later on the lymphatic glands shared in this also. Slight sponginess of the gums, pleural pain, retinal hæmorrhages, and cough were also present. The blood, when counted, showed only 34 per cent. of polynuclear cells, 63 lymphocytes, 2·5 eosinophiles, and a very few mast-cells.

**Chlorosis.**—Leichtenstein[12] discusses the occurrence of venous thrombosis, which is not infrequent in this disease. Œdema may be absent, and the block is generally situated either in the leg or in the cerebral sinuses. In the thrombosis of acute disease we find the femoral vein alone in the lower extremity affected, but in chlorosis the veins of the leg are just as often attacked. Hæmorrhage and pregnancy are frequent predisposing causes. When the clot is deeply situated, with little or no œdema and little pain, there is danger lest friction with liniment should be ordered for supposed myalgia, or exercise be allowed at any stage of the attack, and consequent pulmonary embolism take place. This is curiously frequent in chlorotic thrombosis, so much so that one-fifth of the cases where the legs were affected ended in this manner. It may be useful to note that a high temperature and rigors do not accompany chlorotic thrombosis.

J. Weiss[13] has investigated the most useful form of iron in chlorosis, and reviews the numerous preparations employed in Germany, some thirty in number. He expresses his preference for the albuminate of iron with arsenic in weakly patients, and for iron somatose in those cases especially where there is much gastric irritation and intolerance of drugs, or after some progress has been made upon the albuminate. Hofman[14] has investigated the mode of absorption of iron, and its action. He claims that it is taken up from the duodenum and is in part excreted into the large intestine. It exercises a stimulating effect on the physiological activity of the bone marrow, and quickens the maturity of the young blood corpuscles formed in it. Chlorosis, he believes, is a transitory diminution of the productiveness of the blood-forming organs, especially of the marrow, frequently occurring at puberty, but in some instances it is due to a congenital deficiency.

**Addison's Disease.**—The diagnosis of atypical cases in which there is little or no pigmentation is very difficult, but the disease may be suspected, says Byrom Bramwell, when marked and progressive asthenia without emaciation, fever, or the apparent cause developes, particularly in a young person. Whenever it can be found pigmentation of the mucous membranes is of the highest value. Occasionally pigmentation appears at an early stage, when there are no constitutional symptoms such as weakness or vomiting, and here the difficulties of diagnosis may be insuperable. H. Colman[16] records an instance where, with patches of pigment, there were seen also patches of leucoderma, as though the disease which caused an increase of the colouring matter in one spot had lessened it in another. The patient, a groom, aged 40, complained of great weakness and loss of flesh, and finally suffered from diarrhœa and uncontrollable vomiting. The skin generally was of a dirty brown appearance, and on the lips were patches of even darker pigment. On the face, hands, knees, and abdomen were patches of pure white; while around them the pigmentation was darker than elsewhere, as if the colour had been carried out and heaped up all around. During the last weeks of his life these increased rapidly in size. It may be added that tuberculous disease of the suprarenals and peritoneum was discovered post-mortem. The presence of great abdominal tenderness, together with vomiting, is regarded by Ebstein[17] as an important symptom in Addison's disease, especially where pigmentation is absent. In some of these cases the least touch on the abdomen gives much pain, and the abdominal muscles are strongly contracted, so that the condition is spoken of as "pseudo-peritonitis." Still there is an absence of fever, and the bowels act regularly. The properties of the suprarenal extract, or epinephrin, are becoming better known. B. Moore[18] points out that the astonishing rise of blood pressure it causes when injected subcutaneously is due to direct action on the muscular coats of the arterioles, and not to any effect on the heart or the vasomotor nerves. He had been inclined to regard the substance as a reduced pyridin, but the action differed from that seen in substances of that class. The therapeutic use of the extract presents some difficulties. In the first place, it very easily goes bad, and when taken internally its effects are very uncertain. L. Howe advises that a solution of it should be first heated to 160 deg., and then have a little boric acid added, or a 35 per cent. glycerin solution should be made, or a paste of the extract should be formed into wafers, and, finally, he suggests that it may be preserved by formalin of the strength of

1 in 10,000. All these preparations are, however, somewhat irritating. W. H. Bates, in the same discussion, remarked that there was no dilatation after the contraction caused by the drug. He also showed tracings to prove that it was a cardiac stimulant, and that when applied to the eye it might be absorbed so freely as to actually cause greater strength and regularity of the pulse. Other speakers referred to its value in asthma due to nasal obstruction, as a hæmostatic in nasal surgery, in acute tonsillitis, œdema of the glottis, in stricture of the urethra to facilitate the passage of catheters, and even in acute gonorrhœa and painful micturition. In bleeding from the nose, in hæmophiliacs, and some forms of hay fever it has frequently brought about satisfactory results.

[11] Lancet, Mar. 13. [12] Brit. Physic., Mar. 15. [13] Clinic Excerpts, Feb. [14] Med. Chron., Nov. [15] Clinic Jour., Jan. 17. [16] Lancet, Feb. 17. [17] La Semaine Med., Dec. 13. [18] Med. Rec., Mar. 17.

